# A Real‐Time, Location‐Aware Remote Patient Monitoring System for Telemedicine Decision Support

**DOI:** 10.1155/ijta/6673101

**Published:** 2026-04-17

**Authors:** Adriana-Lavinia Cioca, Marius Cioca, Bogdan Neamtu

**Affiliations:** ^1^ Department of Clinical Medicine, Faculty of Medicine, Lucian Blaga University of Sibiu, Sibiu, Romania, ulbsibiu.ro; ^2^ CMI Cioca Adriana Lavinia, Sibiu, Romania; ^3^ Department of Industrial Engineering and Management, Faculty of Engineering, Lucian Blaga University of Sibiu, Sibiu, Romania, ulbsibiu.ro; ^4^ Faculty of Medicine, Lucian Blaga University of Sibiu, Sibiu, Romania, ulbsibiu.ro; ^5^ Pediatric Clinical Hospital, Sibiu, Romania

**Keywords:** location-aware monitoring, mobile health (mHealth), real-time patient monitoring, remote healthcare, risk-based alerting, telemedicine, wearable sensors

## Abstract

Telemedicine has become an integral component of modern healthcare, particularly in contexts where direct patient–physician interaction is limited or unavailable. In such situations, access to objective physiological data, real‐time patient localization, and automated alerting can significantly improve situational awareness and support timely clinical decision‐making. This paper presents a real‐time, location‐aware patient monitoring system developed as a low‐cost, fully functional demonstration platform to support telemedicine services and risk‐based alerting. The proposed system combines wearable‐based physiological data acquisition with a mobile application that collects heart rate measurements and geographic coordinates, which are transmitted to a server‐side platform for real‐time processing, visualization, and alert generation. Patient status is displayed through an interactive map with risk‐level indicators and complemented by time‐series charts that facilitate the interpretation of physiological trends over time. Incoming data are continuously evaluated using a rule‐based risk assessment mechanism, enabling automated email alerts when predefined critical conditions are detected. Alert notifications include relevant physiological values together with direct links to the patient′s geographic location, supporting rapid response in emergency or high‐risk scenarios. The system is evaluated from a functional and architectural perspective, demonstrating its ability to support remote monitoring, contextual awareness, and decision support in telemedicine settings, including telephone‐based consultations. While the platform does not aim to provide clinical validation or long‐term medical assessment, it illustrates the practical benefits of integrating wearable data, real‐time localization, and automated alerting within a unified telemedicine‐oriented framework. In addition, the proposed architecture is designed to support future extensions based on data‐driven methods, including machine learning–based risk prediction and preventive health analytics. However, such approaches are not part of the current implementation and are outlined as directions for future research. The main contribution of this work lies in the design and implementation of a low‐cost, location‐aware telemedicine monitoring system that combines real‐time data acquisition, integrated visualization, and actionable alerting within a unified and deployable architecture.

## 1. Introduction

Telemedicine has experienced substantial growth in recent years, driven by advances in mobile technologies, wearable devices, and network connectivity, as well as by the increasing demand for remote healthcare services. Its importance became particularly evident during periods when face‐to‐face medical consultations were limited or unavailable, highlighting the need for reliable tools that enable clinicians to assess patient conditions from a distance [1–3]. In many real‐world settings‐especially in primary care and emergency contexts‐telemedicine interactions continue to rely heavily on telephone communication, where clinical decisions are often made based primarily on subjective information reported verbally by patients.

A central challenge of telephone‐based telemedicine lies in the limited availability of objective, real‐time physiological data and contextual information. Without direct observation or access to vital signs, clinicians may encounter difficulties in accurately assessing the severity of a patient′s condition, potentially leading to delayed interventions or overly cautious decision‐making [4, 5]. These limitations underscore the need for systems that complement verbal communication with continuous physiological monitoring, real‐time data visualization, and automated risk assessment.

Wearable devices and mobile health (mHealth) technologies have emerged as key enablers in addressing these challenges. Contemporary wearable sensors are capable of continuously capturing physiological parameters such as heart rate (HR), oxygen saturation, and physical activity, while smartphones provide the necessary computational resources, network connectivity, and access to location services [6–8]. When integrated into telemedicine platforms, these technologies can offer clinicians a more comprehensive and objective view of a patient′s condition, even when the patient is geographically distant.

Beyond physiological monitoring, patient localization represents a critical yet often underexplored dimension of telemedicine systems. In emergency or high‐risk situations, timely knowledge of a patient′s geographic location can substantially reduce response times and facilitate coordination with healthcare providers or emergency services [9]. Location‐aware approaches that combine physiological data with geographic information therefore have the potential to enhance situational awareness and support more informed clinical decision‐making, particularly when rapid action is required.

Automated alerting constitutes another essential component of effective remote patient monitoring. Continuous data streams can easily overwhelm clinicians if not appropriately filtered or prioritized. Risk‐based alert mechanisms that notify healthcare providers when predefined thresholds are exceeded help focus attention on critical cases while minimizing unnecessary interruptions [5]. Among available communication channels, email‐based alerts remain a practical and widely supported solution, enabling the delivery of concise summaries and actionable information, including direct links to patient locations through online mapping services.

Within this context, the present work introduces a real‐time, location‐aware patient monitoring system developed as a functional demonstration platform to support telemedicine services and risk‐based alerting. The system integrates wearable‐derived physiological data‐specifically HR measurements used for demonstration purposes‐with a mobile application that acquires geographic coordinates and transmits the combined data to a server‐side platform. The collected information is visualized using an interactive map with risk‐level indicators and complemented by time‐series charts that allow clinicians to observe physiological trends over time.

The proposed system is not intended to provide clinical diagnosis or long‐term medical validation. Rather, its primary objective is to demonstrate the feasibility and practical value of integrating real‐time physiological monitoring, geographic context, and automated alerting within a unified, telemedicine‐oriented framework. By focusing on system functionality and architectural design, this work illustrates how such an approach can enhance remote clinical decision support, particularly in scenarios where patient assessment relies on telephone‐based communication and timely situational awareness is essential.

Furthermore, the modular architecture of the platform facilitates future extensions, including the integration of additional physiological parameters, large‐scale data analysis, and machine learning‐based predictive models for preventive healthcare. In this respect, the present study contributes to the growing body of telemedicine research by emphasizing practical deployment, system usability, and real‐world applicability rather than purely theoretical or algorithmic performance.

To better highlight the research contribution of this work, the main contributions of the proposed system can be summarized as follows:

• The design and implementation of a low‐cost, real‐time telemedicine monitoring architecture that integrates wearable‐derived physiological data, smartphone‐based geographic localization, server‐side risk assessment, and clinician‐oriented visualization within a unified framework;
• The integration of physiological monitoring with actionable location‐aware alerting, in which high‐risk events are not only detected but also linked to the patient′s real‐time geographic position to support rapid intervention and navigation;
• The development of a practical and deployable demonstration platform specifically oriented toward telemedicine scenarios in which patient assessment may rely on remote interaction, including telephone‐based consultations;
• The provision of a modular system architecture that supports future extension toward multimodal monitoring and predictive analytics, while maintaining transparency, low implementation complexity, and ease of deployment.

Overall, the central contribution of this work lies in demonstrating how real‐time physiological monitoring, geographic contextualization, and actionable alerting can be integrated into a low‐cost, lightweight, and deployable telemedicine system. Rather than focusing on isolated components, the proposed approach emphasizes system‐level integration and practical usability in real‐world remote care scenarios.

## 2. Materials and Methods

### 2.1. System Overview

The proposed system was developed as a functional demonstration platform for real‐time patient monitoring and telemedicine support. Its architecture follows a modular client‐server design that integrates wearable‐based physiological data acquisition, a mobile application for data aggregation and geographic localization, and a server‐side platform responsible for data processing, visualization, and alert generation.

**Figure 1:**
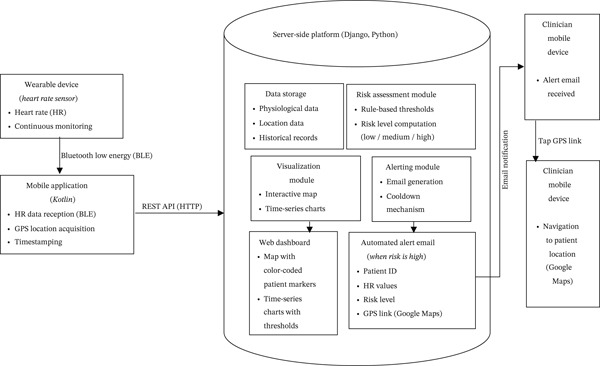
Overall architecture and data flow of the proposed real‐time, location‐aware patient monitoring system.

Figure 1 illustrates the overall architecture of the proposed real‐time, location‐aware patient monitoring system and highlights the complete data flow, from physiological data acquisition to clinician notification and navigation support in high‐risk situations.

All figures presented in this paper are original and were generated using the developed system. User interface elements appear in Romanian, reflecting the localization of the prototype application and its intended use within a real‐world clinical context.

Physiological data are continuously acquired from a wearable HR sensor and transmitted via Bluetooth Low Energy (BLE) to a mobile application, where they are enriched with GPS coordinates and timestamps. The combined data are sent to a server‐side platform for storage, real‐time risk assessment, visualization via a web dashboard, and automated alert generation. When a high‐risk condition is detected, an alert email containing relevant physiological information and a direct link to the patient′s geographic location is sent to the clinician, enabling rapid navigation using an online mapping service.

Following the architecture illustrated in Figure 1, the system supports continuous acquisition of physiological data, contextual enrichment through geographic localization, and centralized processing on the server‐side platform. This design enables real‐time risk evaluation, intuitive visualization, and timely alerting, facilitating both continuous remote monitoring and rapid intervention in high‐risk situations.

The primary objective of the system is to provide clinicians with real‐time access to objective physiological data and geographic context, particularly in telemedicine scenarios where consultations are conducted remotely, including telephone‐based interactions. To support practical deployment, the platform was designed to be lightweight, extensible, and suitable for operation on standard consumer hardware, without requiring specialized medical infrastructure.

### 2.2. Wearable Device and Physiological Data Acquisition

For demonstration purposes, HR was selected as the primary physiological parameter due to its widespread availability in consumer wearable devices and its relevance as an indicator of acute physiological stress. HR data were collected using a generic wearable fitness bracelet capable of continuous HR monitoring. The proposed system does not depend on a specific manufacturer or proprietary cloud ecosystem, allowing flexibility in the choice of wearable devices.

Although HR was used as the primary physiological parameter in this study, this choice was made intentionally to support a low‐cost, accessible, and easily deployable implementation. The system was designed to operate with widely available consumer‐grade wearable devices that do not require proprietary ecosystems, paid services, or complex integration procedures.

Many commercial smartwatches provide additional physiological signals, such as blood oxygen saturation (SpO_2_), body temperature, or activity data, but access to these data is often restricted by vendor‐specific platforms, authentication requirements, or subscription‐based services. In contrast, the proposed approach prioritizes interoperability and independence from closed ecosystems, enabling rapid deployment using simple, affordable devices.

From an architectural perspective, the system is not limited to a single physiological signal. The data acquisition, transmission, storage, and visualization components are designed in a signal‐agnostic manner and can support multiple physiological parameters when such data are available. Therefore, the current implementation should be interpreted as a minimal, cost‐efficient demonstration, while the platform itself remains fully extensible toward multimodal monitoring.

The wearable device communicates directly with the mobile application via BLE, enabling local data acquisition without requiring user accounts or third‐party cloud services. This design choice simplifies deployment, reduces external dependencies, and makes the system suitable for experimental and demonstration‐oriented telemedicine scenarios.

### 2.3. Mobile Application

A custom mobile application was developed for the Android platform using Kotlin (Version 1.9) within Android Studio. The application performs the following core functions:

• Receives HR data from the wearable device via BLE;
• Acquires the smartphone′s geographic coordinates using the built‐in GPS sensor;
• Timestamps incoming data samples;
• Transmits physiological and location data to the server using HTTP‐based RESTful communication.

The mobile application operates in the background and is designed to minimize user interaction, enabling continuous monitoring once user consent has been granted. Geographic coordinates are obtained directly from the mobile device, ensuring accurate localization even when wearable devices do not provide native GPS functionality.

### 2.4. Server‐Side Platform

The server‐side component of the system was implemented using the Django web framework (Version 5.x) running on Python 3.11. Django was selected for its robustness, scalability, and built‐in support for database management and RESTful APIs.

Physiological and location data received from the mobile application are stored in a relational database and processed in real time. Each incoming data sample is evaluated using a rule‐based risk assessment mechanism that compares physiological values against predefined warning and critical thresholds. Based on this evaluation, patients are assigned dynamic risk levels (e.g., low, medium, and high), which are subsequently used for visualization and alerting.

This server‐side design aligns with established principles in biomedical informatics and mHealth data management for handling continuous patient‐generated data streams, including reliable storage, processing, and interoperability considerations [10, 11].

### 2.5. Data Visualization

The system provides two complementary visualization components to support rapid interpretation of patient status:

#### 2.5.1. Map‐based visualization

Patient locations are displayed on an interactive web‐based map, with markers color‐coded according to the current risk level. This representation supports rapid situational awareness and is particularly relevant in emergency scenarios where patient localization is critical.

#### 2.5.2. Time‐series charts

Physiological parameters are displayed as time‐series plots, allowing clinicians to observe trends and variations over selectable time intervals. Charts were implemented using Chart.js, a lightweight JavaScript visualization library suitable for real‐time web applications. Warning and critical threshold lines are overlaid on the charts, together with the mean value of the observed data, providing immediate visual context for interpreting physiological changes.

### 2.6. Risk Assessment and Alerting Mechanism

Risk assessment is performed using a rule‐based approach in which physiological values are compared against predefined thresholds derived from general clinical guidelines. When a high‐risk condition is detected, the system automatically generates an alert.

It is important to note that the current rule‐based risk assessment mechanism is intentionally simplified and designed for demonstration and transparency purposes. The use of fixed thresholds allows clear interpretability and predictable system behavior, which is particularly relevant in early stage telemedicine prototypes and deployment‐oriented platforms.

However, this approach does not account for contextual factors such as physical activity, individual baseline variability, or patient‐specific conditions. For example, elevated HR values during exercise may not necessarily indicate a high‐risk situation. These limitations are acknowledged and represent an important motivation for future extensions toward context‐aware and data‐driven risk assessment models.

For clarity, Algorithm 1 summarizes the runtime logic used by the server to process incoming samples, update the dashboard, and trigger high‐risk alerts.

**Algorithm 1:**
Real‐time monitoring, risk assessment, and alerting workflow.
**Inputs:**
HR(t): heart rate sample received at time *t*
GPS(t): geographic coordinates *(latitude, longitude)* acquired at time *t*
*patient_id*: unique identifier of the monitored patient
TH_warn_low, TH_warn_high: warning thresholds
TH_crit_low, TH_crit_high: critical thresholds
COOLDOWN_MIN: minimum time interval (in minutes) between consecutive alerts for the same patient
**State (per patient)**
*last_alert_time*: timestamp of the most recent alert (initially null)
**Procedure:** *ON_NEW_SAMPLE (patient_id, HR(t), GPS(t), t)*
**1. Data persistence**
  Store the incoming physiological sample and associated geographic coordinates: store_sample(patient_id, HR(t), GPS(t), t)
**2. Rule-based risk evaluation**
  Determine the current risk level based on predefined thresholds:
  o. if HR(t) < TH_crit_low or HR(t) > TH_crit_high, then *risk* ← HIGH
  o. else if HR(t) < TH_warn_low or HR(t) > TH_warn_high, then *risk* ← MEDIUM
  o. else *risk* ← LOW
Update the patient′s current risk status:
update_current_risk(patient_id, risk, t)
**3. Dashboard update**
  Refresh visualization components to reflect the new data sample:
  o. update the patient′s map marker according to current location and risk level
  o. append the heart rate sample to the corresponding time‐series visualization
**4. Alert generation with cooldown control**
  If the detected risk level is HIGH, check whether an alert can be issued:
  o. if *last_alert_time* is null or the elapsed time since the last alert exceeds COOLDOWN_MIN, then
    ▪ compose an alert email including patient identifier, physiological values, timestamp, and a direct geographic link
    ▪ send the alert to the clinician
    ▪ update *last_alert_time* ← *t*

Alerts are delivered via email using Django′s SMTP‐based email backend, as part of the server‐side alerting mechanism described in Algorithm 1. Each alert message contains concise and actionable information, including

• Patient identification;
• Relevant physiological values triggering the alert;
• The timestamp of the event;
• A direct hyperlink to the patient′s geographic location provided through an online mapping service.

Email was selected as the alert delivery channel due to its simplicity, wide availability, and ease of integration within a prototype system. While email‐based notifications may not be optimal for time‐critical emergency response, they provide a reliable and easily deployable mechanism for delivering structured alert information, including patient data and geographic links.

The current implementation should therefore be interpreted as a baseline alerting solution. In real‐world deployments, the system architecture can be readily extended to support faster notification channels, such as push notifications, SMS, or integration with dedicated clinical alerting systems, depending on the urgency and operational requirements.

To prevent excessive notifications and reduce alert fatigue, alert generation is constrained by a cooldown mechanism that limits the delivery to a maximum of one email per patient within a predefined time interval.

### 2.7. Ethical Considerations and Scope

The proposed system is intended solely as a demonstration and proof‐of‐concept platform. No clinical trials or long‐term medical studies were conducted as part of this work, and the system does not provide diagnostic, prognostic, or therapeutic recommendations. All data used during system development and testing were collected with informed consent.

The platform was designed with extensibility and future research in mind. Potential extensions include clinical validation studies and the integration of machine learning–based predictive analytics, subject to appropriate ethical approval and compliance with relevant regulatory requirements.

## 3. Results

The proposed system was evaluated from a functional and operational perspective, with a focus on its ability to collect, process, visualize, and generate alerts based on real‐time patient data. The results presented in this section describe the observed system behavior in realistic telemedicine scenarios, with emphasis on usability, responsiveness, and situational awareness rather than clinical validation.

### 3.1. Real‐Time Data Acquisition and Processing

During system operation, physiological data transmitted from the wearable device via the mobile application were received and processed by the server in real time. Each incoming data sample was automatically associated with a timestamp and corresponding geographic coordinates, enabling continuous monitoring of both physiological status and patient location.

Upon receipt of new data, the server‐side platform dynamically updated patient records and recalculated the associated risk level based on predefined thresholds. Changes in physiological parameters were immediately reflected in both the visualization and alerting components of the system, ensuring timely representation of the patient′s current status.

### 3.2. Map‐Based Visualization of Patient Status

The geographic distribution of monitored patients was displayed using an interactive map interface. Each patient was represented by a marker positioned at the most recent geographic coordinates reported by the mobile application.

**Figure 2:**
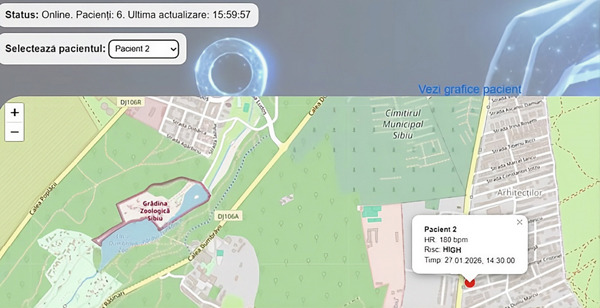
Real‐time map‐based visualization of patient locations. Patients are represented by color‐coded markers indicating the current risk level (green: low risk, orange: medium risk, and red: high risk). The map provides spatial context that supports rapid situational awareness in telemedicine and emergency scenarios. Map data from Google Maps.

Figure 2 presents an example of the map‐based visualization, in which marker colors represent the current risk level: Green markers correspond to low‐risk conditions, orange markers indicate medium‐risk conditions, and red markers denote high‐risk situations requiring immediate attention. This visualization enabled rapid identification of patients in critical states and provided essential spatial context, which is particularly relevant in emergency and telemedicine scenarios where clinicians may not have direct visual contact with the patient.

### 3.3. Time‐Series Visualization of Physiological Parameters

In addition to the map‐based view, the system provided time‐series charts for the monitored physiological parameters. These charts enabled the inspection of temporal trends and fluctuations over selectable time intervals, such as recent hours or days.

**Figure 3:**
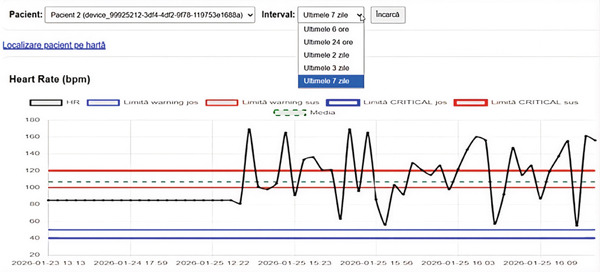
Heart rate time‐series visualization with risk thresholds. Measured heart rate values are shown as a continuous black line. Thin horizontal lines indicate warning thresholds, while thicker horizontal lines denote critical thresholds. The green dashed line represents the mean heart rate over the selected time interval.

Figure 3 presents an example of an HR time‐series chart generated by the system. The visualization incorporates multiple graphical elements, including a continuous line representing measured HR values, horizontal lines indicating warning thresholds, thicker horizontal lines marking critical thresholds, and a dashed line denoting the mean HR over the selected interval. Together, these elements allow rapid assessment of whether observed values remain within normal ranges, approach warning limits, or exceed critical thresholds.

### 3.4. Risk Assessment and Alert Generation

When incoming physiological data exceeded predefined critical thresholds, the system classified the corresponding patient state as high risk and automatically generated an alert.

**Figure 4:**
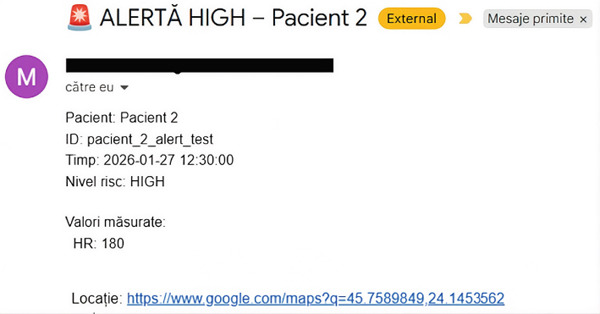
Example of an automated high‐risk alert email generated by the system. The alert message includes patient identification, the physiological values triggering the alert, the timestamp of the event, and a direct hyperlink to the patient′s geographic location provided through an online mapping service.

Figure 4 presents an example of an alert email generated during system operation. Each alert message contained concise and actionable information, including the patient identifier, the physiological values triggering the alert, the timestamp of the event, and a direct hyperlink to the patient′s geographic location provided through an online mapping service. The inclusion of the location link enabled immediate navigation to the patient′s position, supporting timely response by healthcare providers or emergency services.

To prevent repeated notifications for the same patient and reduce alert fatigue, alert generation was constrained by a predefined cooldown interval.

### 3.5. Telemedicine Use Case Scenarios

The system was applied in telemedicine scenarios involving telephone‐based consultations. In these cases, clinicians were able to access real‐time physiological data, historical trends, and patient location information while communicating verbally with the patient. This combination of objective measurements and contextual information supported enhanced situational awareness during remote interactions.

The observed results indicate that integrating wearable‐derived physiological data, real‐time visualization, and automated alerting within a unified platform enables more comprehensive remote patient monitoring, even when patient‐clinician interaction is limited to voice‐based communication.

### 3.6. Technical Performance Evaluation

In addition to functional validation, the system was evaluated from a technical performance perspective, focusing on key operational metrics relevant to real‐time telemedicine applications.

#### 3.6.1. Response Delay

The time between physiological data acquisition on the wearable device and its visualization on the server‐side dashboard was observed to be within a range of approximately 1–3 s under normal network conditions. This delay includes Bluetooth transmission, mobile processing, network communication, and server‐side updates.

#### 3.6.2. Transmission Stability

During continuous operation, the system maintained stable data transmission without significant packet loss in standard mobile network conditions. Temporary interruptions (e.g., due to network fluctuations) were handled by the mobile application through automatic reconnection, ensuring continuity of monitoring.

#### 3.6.3. Alert Latency

The delay between the detection of a high‐risk condition and the delivery of the corresponding alert email was typically below 5 s, depending on network conditions and email server response time. This latency was considered acceptable for the demonstration scope of the system.

#### 3.6.4. GPS Accuracy

Geographic coordinates obtained from the smartphone GPS sensor provided sufficient accuracy for telemedicine use cases, typically within a few meters in outdoor environments. Accuracy may decrease in indoor or signal‐obstructed environments, which is consistent with standard GPS limitations.

Overall, the observed performance indicates that the system is capable of supporting near real‐time monitoring and alerting in practical telemedicine scenarios.

## 4. Discussion

The proposed system follows a design philosophy aligned with previously developed modular eHealth platforms, such as ROboMC, which emphasize the integration of validated data processing pipelines with AI‐assisted components to support reliability, transparency, and adaptability in real‐world medical and educational contexts [12]. Consistent with this perspective, the present work prioritizes system functionality, architectural clarity, and practical deployment considerations, rather than clinical outcome validation.

The results presented in this study demonstrate the feasibility and practical utility of a real‐time, location‐aware patient monitoring system designed to support telemedicine scenarios. A distinguishing aspect of the proposed platform is that its contribution lies not in introducing a new isolated sensing or alerting technology, but in the operational integration of several components into a practical telemedicine‐oriented decision‐support workflow. In particular, the system combines real‐time physiological monitoring, geographic contextualization, visual risk stratification, and actionable clinician alerts in a lightweight architecture designed for rapid deployment using widely available consumer technologies. This deployment‐oriented perspective is particularly relevant for telemedicine settings in which simplicity, low cost, and operational usability are essential. Rather than aiming to demonstrate clinical efficacy, the discussion focuses on the functional benefits of the system, its system‐level implications, and its potential contribution to improving situational awareness and supporting remote healthcare delivery.

To better position the proposed system relative to existing telemedicine solutions, a comparative overview is presented in Table 1.

**Table 1:** Comparison of the proposed system with representative telemedicine monitoring platforms.

Feature/system	Proposed system	Typical wearable cloud systems	Advanced telemedicine platforms
Physiological data	Heart rate (extensible to multisignal monitoring)	Multisignal (HR, SpO_2_, etc.)	Multisignal
Location awareness	Integrated real‐time GPS	Rarely integrated	Sometimes available
Alerting mechanism	Real‐time, rule‐based, location‐aware alerts with direct map links	Notifications within proprietary apps	Complex alert systems
Ecosystem dependency	No (direct device‐to‐app communication)	Yes (vendor cloud platforms)	Often integrated in institutional systems
Deployment cost	Low (consumer‐grade devices, no subscriptions)	Medium to high	High (clinical infrastructure required)
Ease of deployment	High (lightweight architecture)	Medium (account and cloud setup required)	Low (complex integration)
Target scenario	Telemedicine, including phone‐based consultations	Personal health tracking	Clinical monitoring environments
System focus	Integration + actionable alerts + deployability	Data collection and visualization	Clinical data management and analytics

The comparison highlights that the novelty of the proposed system lies not in introducing new sensing technologies, but in the integration of physiological monitoring, geographic context, and actionable alerting within a lightweight and deployment‐oriented architecture. In contrast to many existing solutions that rely on proprietary ecosystems or complex clinical infrastructures, the proposed platform emphasizes accessibility, interoperability, and practical usability in real‐world telemedicine scenarios.

One of the primary strengths of the proposed platform lies in its ability to complement telephone‐based telemedicine consultations with objective physiological data and real‐time contextual information. In many healthcare settings—particularly in primary care and emergency triage—clinicians often rely on verbal descriptions of symptoms provided by patients. Such descriptions may be incomplete, subjective, or influenced by patient anxiety. By providing continuous access to physiological measurements and historical trends, the system enhances situational awareness and supports more informed decision‐making during remote consultations.

The integration of real‐time geographic localization represents a significant enhancement compared to many existing remote monitoring solutions, which typically focus exclusively on physiological parameters. In emergency or high‐risk situations, rapid access to a patient′s geographic location can reduce response times and facilitate coordination with emergency services. The inclusion of direct map‐based links within alert notifications further strengthens this capability by transforming alerts from passive warnings into actionable, context‐aware information.

Automated, risk‐based alerting constitutes another important contribution of the proposed system. Continuous monitoring can generate large volumes of data, potentially overwhelming healthcare providers if not appropriately filtered. The rule‐based risk assessment mechanism implemented in this work illustrates how predefined thresholds can be used to prioritize critical situations and trigger alerts selectively. Although intentionally simple, this approach aligns with established early warning practices and proved sufficient for demonstrating real‐time alerting behavior within a telemedicine context.

The visualization components of the platform—namely, the interactive map and time‐series charts—also play a central role in system usability. The combined use of spatial and temporal representations allows clinicians to rapidly interpret patient status, identify abnormal trends, and relate physiological changes to geographic context. The inclusion of warning and critical thresholds, together with mean value indicators, provides immediate visual cues and reduces the cognitive effort required to interpret raw numerical data during remote consultations.

Despite these strengths, several limitations of the current system should be acknowledged. First, the platform was developed as a proof‐of‐concept rather than a clinically validated medical device. The evaluation relied primarily on HR measurements, selected for their availability in consumer wearable devices and their relevance as indicators of acute physiological stress. While informative, HR alone does not capture the full complexity of a patient′s health status.

In particular, the use of fixed threshold‐based rules represents a simplified decision mechanism that does not capture the full complexity of clinical reasoning. In real‐world scenarios, clinicians interpret physiological signals in relation to context, including patient activity, medical history, and temporal patterns. The current implementation does not incorporate such contextual awareness and may therefore generate alerts in situations that are not clinically critical, such as during physical exertion.

Nevertheless, the use of transparent rule‐based logic provides a clear and interpretable baseline that is suitable for demonstration purposes and system validation. This design choice also facilitates future comparison with more advanced approaches, such as machine learning–based predictive models or context‐aware risk assessment strategies.

Second, the risk assessment mechanism is based on predefined rule‐based thresholds derived from general clinical guidelines. Although this approach offers transparency and interpretability, it does not account for individual patient variability, long‐term trends, or contextual factors such as comorbidities or medication use. Consequently, the current system is best suited for demonstrating functional capabilities and architectural feasibility, rather than providing personalized medical assessments.

The use of a single physiological signal should also be understood in the context of system accessibility and deployment constraints. By relying on a simple, low‐cost wearable device that provides HR data without requiring proprietary cloud ecosystems, the system demonstrates that meaningful telemedicine monitoring can be achieved with minimal technical and financial barriers.

At the same time, the proposed architecture remains fully compatible with multimodal monitoring. When devices capable of providing additional physiological signals are available, these can be integrated without modifying the core system design. This flexibility enables a gradual transition from minimal, cost‐efficient deployments toward more advanced and clinically comprehensive monitoring solutions.

These limitations also point toward several promising directions for future research. The modular design of the platform enables the integration of additional physiological parameters, such as oxygen saturation, body temperature, and activity‐related metrics, as well as the incorporation of external datasets for comparative analysis. More importantly, the system provides a solid foundation for the integration of machine learning techniques aimed at predictive risk assessment and preventive healthcare.

Although machine learning is discussed as a potential extension, it is not included in the current implementation. This choice reflects the focus of the study on system integration and real‐time operational functionality. At this stage, we intentionally avoided introducing a superficial machine learning component without clinically validated labeled data, in order to maintain methodological clarity and avoid overstating the capabilities of the system. The proposed architecture, however, is designed to support future incorporation of data‐driven models without requiring fundamental changes to the system design.

The technical performance evaluation further supports the practical applicability of the system, demonstrating that real‐time data transmission, visualization, and alerting can be achieved with low latency using standard consumer hardware and network infrastructure. These results reinforce the suitability of the proposed approach for deployment‐oriented telemedicine scenarios.

Future work may explore data‐driven models capable of learning patient‐specific patterns and estimating the likelihood of high‐risk events before they occur. By analyzing temporal windows of physiological data, such models could support a transition from reactive alerting toward proactive prevention. In this context, the current rule‐based framework can serve both as a baseline for comparison and as a source of pseudolabels during early stages of supervised learning.

From a broader telemedicine perspective, the proposed platform highlights the value of integrating monitoring, localization, and alerting within a unified framework. Rather than replacing clinical judgment, such systems can function as decision‐support tools that enhance remote consultations, particularly in situations where direct visual assessment is not possible. As telemedicine continues to expand, the integration of real‐time physiological and contextual data is likely to play an increasingly important role in improving care quality and patient safety.

Finally, this work builds upon previous research on low‐cost, deployable AI‐based healthcare systems, such as the visionMC platform, which demonstrated the feasibility of supporting primary care workflows, patient identification, and automated alerting with minimal infrastructure requirements [13]. While visionMC focused primarily on clinic‐level workflow optimization, the proposed system extends this paradigm to continuous patient monitoring and location‐aware telemedicine support.

## 5. Future Research Directions

While the current system demonstrates the feasibility and practical benefits of real‐time, location‐aware patient monitoring for telemedicine support, several research directions can further enhance its capabilities and clinical relevance. These directions focus on extending the platform from a primarily reactive monitoring solution toward a more predictive and preventive healthcare framework.

### 5.1. Integration of Additional Physiological Parameters

An immediate direction for future research involves expanding the range of monitored physiological parameters. Although HR was selected as the primary signal for demonstration purposes, additional data such as SpO_2_, body temperature, physical activity levels, and sleep‐related metrics could be integrated into the platform. The inclusion of multimodal physiological data would enable a more comprehensive assessment of patient status and provide richer input for advanced analytical methods.

Combining multiple physiological signals may also improve system robustness by reducing reliance on a single parameter and mitigating noise or artifacts commonly associated with wearable sensors. Future studies may explore the relative contribution of individual parameters to risk assessment and investigate optimal feature combinations for specific telemedicine use cases.

### 5.2. Machine Learning–Based Risk Prediction

A key research direction involves transitioning from rule‐based risk assessment to machine learning–based predictive modeling. While predefined thresholds offer transparency and simplicity, they are inherently limited in their ability to capture complex temporal dynamics and interindividual variability. Machine learning models trained on historical physiological data could learn patient‐specific baselines and identify subtle deviations indicative of emerging high‐risk conditions.

Such models may operate on sliding time windows (e.g., recent minutes of physiological data) to estimate the likelihood of adverse events within a predefined prediction horizon. This predictive approach would enable earlier intervention and shift the system′s role from reactive alerting toward proactive prevention. Importantly, the existing rule‐based framework can serve as an initial reference during early experimentation, providing baseline comparisons or pseudolabels for supervised learning until clinically annotated datasets become available.

At this stage, machine learning components are not integrated into the current system implementation. The focus of the present work is on system architecture, real‐time data integration, and deployment‐oriented design. However, the existing platform provides a suitable foundation for future experimentation with data‐driven models, as it already supports structured data collection, storage, and real‐time processing pipelines required for such approaches.

### 5.3. Anomaly Detection and Personalized Baselines

In addition to supervised prediction, unsupervised or semisupervised anomaly detection techniques represent a promising avenue for future research. By modeling normal physiological behavior at the individual level, anomaly detection algorithms could identify atypical patterns that may not exceed predefined thresholds but still warrant clinical attention.

This personalized approach is particularly relevant in telemedicine settings, where patient populations are heterogeneous and standard thresholds may not be appropriate for all individuals. Integrating anomaly detection with predictive models could result in hybrid systems that combine population‐level trends with patient‐specific insights.

### 5.4. Dataset Expansion and External Data Integration

Another important research direction concerns the expansion of datasets used for model development and evaluation. While the current platform focuses on real‐time data collected during system operation, future work may incorporate publicly available physiological datasets to augment training data and improve model generalization.

External datasets could support benchmarking studies, transfer learning approaches, and evaluations of how models trained on controlled datasets perform when applied to real‐world, wearable‐derived data. Such efforts would enhance reproducibility and facilitate meaningful comparisons across different telemedicine platforms.

### 5.5. Clinical Validation and Ethical Considerations

For the system to evolve beyond a demonstration platform, future research must address clinical validation and ethical considerations. This includes collaboration with healthcare professionals to define clinically meaningful endpoints, assess alert relevance, and evaluate the impact of predictive models on clinical workflows.

Ethical aspects such as data privacy, informed consent, and transparency of algorithmic decision‐making remain critical. As analytical models become more complex, ensuring explainability and maintaining clinician trust will be essential for successful adoption in real‐world telemedicine environments.

### 5.6. Toward Preventive and Decision‐Support Systems

Ultimately, the integration of advanced analytics and machine learning has the potential to transform the proposed platform into a preventive decision‐support system. By identifying early warning signs and contextual risk factors, such systems could assist clinicians in prioritizing follow‐up actions, tailoring interventions, and allocating healthcare resources more effectively.

Rather than replacing medical expertise, future iterations of the platform are aimed at augmenting clinical judgment by providing timely, data‐driven insights within established telemedicine workflows. This evolution aligns with broader trends in digital health, where intelligent systems increasingly support clinicians in delivering safer, more efficient, and more personalized care.

From a system‐level perspective, the main contribution of this work is the integration of monitoring, localization, and alerting into a coherent and deployment‐oriented telemedicine framework. This unified approach distinguishes the proposed system from solutions that focus primarily on isolated functionalities without emphasizing real‐world usability and rapid deployment.

## 6. Conclusions

This paper presented a real‐time, location‐aware patient monitoring system developed as a functional demonstration platform to support telemedicine services and risk‐based alerting. The proposed solution integrates wearable‐derived physiological data, mobile‐based geographic localization, and server‐side processing within a unified architecture aimed at enhancing situational awareness and decision support in remote healthcare scenarios.

The system addresses a practical and increasingly relevant challenge in telemedicine, particularly in situations where consultations are conducted without direct visual contact between patients and clinicians. By complementing verbal communication with objective physiological measurements, historical trend visualization, and real‐time location information, the platform provides clinicians with a more robust basis for patient assessment during remote interactions. Automated, risk‐based alerting further supports timely intervention by highlighting critical situations without requiring continuous manual supervision.

From a functional perspective, the system demonstrated reliable real‐time data acquisition, dynamic risk evaluation, and intuitive visualization through both map‐based interfaces and time‐series charts. The alerting mechanism, which delivers concise summaries and direct geographic links via email, transforms physiological events into actionable information and supports rapid response in emergency or high‐risk situations. Together, these components illustrate the benefits of integrating monitoring, localization, and alerting within telemedicine‐oriented systems.

It is important to note that the proposed platform was developed as a proof of concept rather than a clinically validated medical device. The evaluation focused on system functionality and architectural feasibility, using HR data collected from a generic wearable device for demonstration purposes. No diagnostic or therapeutic claims are made, and the results should be interpreted within this experimental context. Nevertheless, the system demonstrates how readily available consumer technologies can be combined to address real‐world telemedicine needs in a practical and scalable manner.

The modular design of the platform provides a flexible foundation for future extensions. Potential developments include the integration of additional physiological parameters, the incorporation of larger and more diverse datasets, and the application of machine learning techniques for predictive risk assessment and preventive healthcare. Such extensions could enable a transition from reactive monitoring toward proactive decision support, further enhancing the clinical relevance of the system.

In conclusion, this work shows that a real‐time, location‐aware monitoring platform can effectively support telemedicine by providing objective data, contextual awareness, and automated alerts within a unified framework. By emphasizing practical deployment and usability, the proposed system contributes to the advancement of digital health solutions aimed at supporting clinicians and improving patient safety in remote care environments.

## Funding

No funding was received for this manuscript.

## Conflicts of Interest

The authors declare no conflicts of interest.

## Data Availability

The data supporting the findings of this study were generated during the development and testing of a proof‐of‐concept system and are not publicly available due to privacy and ethical considerations. Aggregated or anonymized data may be made available from the corresponding author upon reasonable request.
